# Exploring Food Literacy Domains in an Adult Samoan Population

**DOI:** 10.3390/ijerph18073587

**Published:** 2021-03-30

**Authors:** Grace Kammholz, Dana Craven, Ramona Boodoosingh, Safua Akeli Amaama, Jyothi Abraham, Sarah Burkhart

**Affiliations:** 1 School of Health and Behavioural Sciences ML41, University of the Sunshine Coast, Locked Bag 4, Maroochydore DC, QLD 4558, Australia; glk004@student.usc.edu.au (G.K.); dcraven1@usc.edu.au (D.C.); 2 Australian Centre for Pacific Islands Research, University of the Sunshine Coast, Locked Bag 4, Maroochydore DC, QLD 4558, Australia; 3 Faculty of Health Science, School of Nursing, National University of Samoa, Apia, Western Samoa; j.abraham@nus.edu.ws; 4 Centre for Samoan Studies, National University of Samoa, Apia, Western Samoa; safua.akeli.amaama@tepapa.govt.nz

**Keywords:** Pacific Islands, food knowledge, food skills, food behaviours, food choice, nutrition, health

## Abstract

Samoan food systems have undergone a dramatic nutrition transition, with dietary patterns changing concurrently with increased rates of obesity and non-communicable disease. Whilst policy action and environmental interventions play an important role in improving access to and consumption of healthy food, the success of these relies on a greater understanding of individuals’ food knowledge and behaviours. This study aimed to explore these behaviours using the construct of food literacy in an adult Samoan population. A cross-sectional interviewer-administered questionnaire of a convenience sample of 150 adult Samoans (≥20 years) assessed the four domains of food literacy: plan/manage, select, prepare, and eat. Participants generally plan to include healthy food (87%) and budget money for food (87%). The majority know where to find nutrition labels (68%), of which 43% always use them to inform their food choices. Participants were mostly confident with cooking skills, although food storage practices require further investigation. Over 90% agreed or strongly agreed that food impacts health, although understanding of the Pacific Guidelines for Healthy Living was lacking. Understanding the ability of Samoans to plan/manage, select, prepare, and eat food is an important consideration for future interventions aiming to assist this population in navigating the modern-day food system.

## 1. Introduction

In settings where food environments are rapidly changing, understanding food behaviours has the potential to support healthy, sustainable diets. Food systems are changing throughout Pacific Island Countries (PICs), with this transition significantly impacting food environments and human health [1]. Over the last six decades, diets traditionally characterised by high consumption of subsistence-sourced local staples and fresh foods have shifted to diets reliant on imported, processed foods [2], threatening food security and health [3]. 

Samoa, an archipelago located in the South Pacific Ocean, is facing a health epidemic of rising obesity and diet-related non-communicable diseases (DR-NCD) [4]. In 2016, almost half of the adult population were considered obese and DR-NCD accounted for an estimated 81% of mortality [5]. The Pacific Guidelines for Healthy Living are available for Samoans, which promote the consumption of three key food groups: protective foods (fresh, locally grown fruits and vegetables, such as banana and cabbage), energy foods (e.g., starchy staples such as taro and rice), and bodybuilding foods (protein-rich foods such as fish and eggs) [6].

An improvement in pro-health behaviours, particularly food choice and consumption, could help to reduce the incidence of overweight, obesity, and DR-NCD and consequently reduce pressure on health systems. However, to practice healthy, sustainable dietary behaviours, one must first be equipped with the knowledge and skills required to navigate modern-day food systems and make appropriate food choices. Food literacy is an emerging construct that seeks to describe these practical food-related aspects of day-to-day nutrition [7].

Food literacy is “the scaffolding that empowers individuals, households, communities or nations to protect diet quality through change and strengthen dietary resilience over time. It is composed of a collection of inter-related knowledge, skills and behaviours required to plan, manage, select, prepare and eat food to meet needs and determine intake” [7] (p. 54). The framework for “food literacy” outlined by Vidgen and Gallegos includes components within four domains including to: (i) plan and manage; (ii) select; (iii) prepare; and (iv) eat [7]. These domains encompass aspects of food consumption including meal planning and budgeting (plan and manage), using nutrition information panels to identify healthier options (select), safely preparing and storing food at home (prepare), and understanding which foods are healthier options and which ones to consume more of (eat). Understanding the food literacy behaviours of adults may be useful in education interventions to better support individuals and communities in transitioning food environments.

To date, the knowledge, skills, and behaviours required to plan and manage, select, prepare, and eat have not been well studied in Pacific Islander populations, highlighting a gap in our understanding of how this population makes food decisions. Research has examined the broader concept of health literacy in Samoa. Bollars et al. (2019) conducted a qualitative study using focus group interviews to explore how people access, understand, appraise, and apply information to make decisions around healthcare, disease prevention, and health promotion [8]. Health literacy was found to be influenced by culture and the family circle proved to be central to health. Personal ownership of health was found to be lacking and, despite basic knowledge of NCDs, the population lacked a deeper understanding of chronic disease implications [8]. Despite the established nature of health literacy within the context of public health promotion, food literacy explicitly focuses on health literacy abilities in a food context and, as such, advances health literacy in nutrition interventions [9]. In Samoan populations, elements of food literacy, such as food knowledge and/or education [10,11,12], food cost/budgeting [12,13], and food choice behaviours [14,15], have been explored. However, a framework was not previously used to evaluate their level of food literacy. While this existing work provides an insight into food decisions, exploring food-related behaviours within a framework that encapsulates food literacy is warranted to elucidate a wider breadth of understanding of individual food practices. This work may then underpin potential public health interventions and provide evidence for policymakers. Therefore, this descriptive, exploratory study aims to describe the food literacy of adult Samoans, in the context of the four domains of plan and manage, select, prepare, and eat.

## 2. Materials and Methods

### 2.1. Study Setting and Design

**Figure 1:**
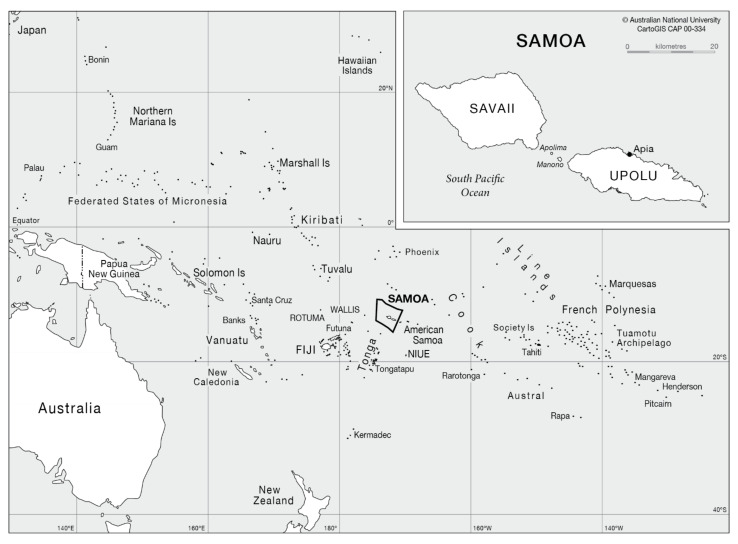
Map of the South Pacific Ocean showing the location of Samoa (formerly known as Western Samoa) and its capital, Apia, from CartoGIS Services, College of Asia and the Pacific, The Australian National University [18].

This cross-sectional study explored food literacy in resident Samoan adults and was part of a larger study examining the food environment in Samoa by a team of five researchers (three from the National University of Samoa (NUS) and two from the University of the Sunshine Coast (USC)) and 12 undergraduate research assistants (11 NUS and one USC) (hereafter known collectively as the research team). The study was undertaken in Samoa, an archipelago located in the South Pacific Ocean, with a total land area of 2830 square kilometres and a population of approximately 192,000 people [16]. The two main inhabited islands are Upolu and Savaii, housing 77% and 22% of the total population, respectively. The rest of the population reside on the islands of Namua, Apolima, and Manono, although these were not included in this study [17]. The capital city, Apia, is located on the smaller island of Upolu (Figure 1).

### 2.2. Participant Recruitment

Participants were recruited from Apia (Upolu island) and Salelologa (Savaii island) over an eight-day period in June 2019. Eligibility criteria included adult (aged ≥ 20 years) Samoan or English-speaking residents. Researchers approached individuals at shopping centres, markets, schools, and bus stations across both locations, inviting them to participate in an interviewer-administered food literacy questionnaire. Interested individuals were provided with an information sheet outlining the project purpose and use of data and then asked to sign an informed consent form if they were willing to participate. The questionnaire was conducted at the place of invitation and participants received WST 10 (the local currency Western Samoa Tala) following completion but were unaware of the incentive at the time of invitation. The study was conducted according to the guidelines of the Declaration of Helsinki and approved by the National University of Samoa Research and Ethics Committee (UREC) (15-11-18-1.1).

### 2.3. Data Collection Tool

A paper-based, interviewer-administered, structured questionnaire was used to collect data from participants. Questions to explore food literacy were embedded into a broader questionnaire (exploring the food environment in Samoa). Food literacy questionnaire items were derived from the literature [19,20,21,22] and categorised into one of the four conceptual food literacy domains: (i) plan and manage, (ii) select, (iii) prepare, or (iv) eat. The tool did not encompass all components of food literacy due to a lack of validated questions being available and potential respondent burden of the long questionnaire. The questions included were defined as being of importance in this population, based on the limited literature in this context and agreed upon by the research team.

The questionnaire was reviewed for content validity and formatting by all members of the research team. National University of Samoa researchers (n = 3) completed an assessment of face validity and provided changes where required for cultural appropriateness. The tool was constructed in English and translated into Samoan by a native Samoan and English speaker. The final questionnaire was made available in Samoan and English, with a final review undertaken by NUS researchers to ensure correct translation. Field testing was completed on day one of data collection, with minor structural changes (i.e., order of questions) made to the questionnaire.

The final questionnaire contained 17 food literacy questions. The following demographic information was collected: gender; age (years); marital status; employment status; level of highest completed education, and current place of residence (village and island).

To explore the domain of (i) plan and manage, participants were asked to rate how frequently they engaged in the following behaviours: plan meals ahead of time, make a list before you go shopping, plan to include healthy food when deciding what to eat, and budget money for food on a 4-point Likert scale (never or rarely, sometimes, most of the time, always, with a non-applicable option).

To explore the domain of (ii) select, participants were asked to answer the questions, “when you go shopping do you know where to locate nutrition information on foods?” (yes/no/I am not sure responses) and “if yes, how often do you use this information to make food choices?”, on a 4-point Likert scale (never or rarely, sometimes, most of the time, always, or I know where it is but I can’t use it as it is in a different language).

The domain of (iii) prepare asked participants to rate how frequently they engaged in the following: cooking meals at home, feeling confident cooking a variety of foods, changing recipes to make them healthier (all on a 4-point Likert scale; never or rarely, sometimes, most of the time, always, with a non-applicable option). Food safety and hygiene questions included “do you know how to store food to make it last longer?” (yes/no/I am not sure responses) and explored level of agreement with the following statements: “you should always wash hands with soap before and after preparing foods” and “cooked foods can be stored at room temperature overnight”. These were rated on a 5-point visual Likert scale from strongly disagree to strongly agree.

The final domain, (iv) eat, was explored through five items. Participants were asked to indicate their level of agreement with the following statements: “food has an impact on my health and eating processed foods that are high in salt and fat, such as canned beef, is harmful to my health” (5-point Likert scale from strongly agree to strongly disagree). Knowledge of the Pacific Guidelines for Healthy Living [6] was explored through the use of three questions: “could you identify the three food groups used in the dietary guidelines from this list?” (responses: protective foods, imported foods, healthy foods, bodybuilding foods, local foods, energy foods, and I am not sure) and, using pictorials, “can you identify which of these are protective foods?” (pictures of taro, rice, banana, fish, cabbage, eggs, and an I am not sure option), and “how often should you eat protective foods?” (once a week to at every meal, with an I am not sure option).

### 2.4. Data Collection

Prior to the commencement of data collection, training was provided for the undergraduate research assistants responsible for administering the questionnaires. This training included role play activities to assist researchers in becoming more comfortable approaching individuals and inviting them to participate, how to ask questions, and recording responses. Laminated, A-4-sized picture charts were used to complement verbal questions to ensure clarity for responses. Pictures of foods were used to aid responses to questions related to food groups and visual Likert scales were also used with thumbs-up icons for strongly agree and thumbs-down for strongly disagree. The research team travelled to shopping areas and primary schools and then worked in pairs, with at least one Samoan-speaking individual, to complete the interviewer-administered questionnaires. At the end of each day, the research team regrouped to discuss data collection activities and address questions and/or concerns.

### 2.5. Data Analysis

Data were entered into Microsoft Excel (Microsoft Office 365) by a trained research assistant. All variables were documented in a codebook and included descriptive labels and numeric codes for categories. Any text written in Samoan was entered into the spreadsheet verbatim and translated to English by a member of the NUS team. Questionnaires requiring translation from Samoan back to English were completed by one Samoan research assistant. To ensure accuracy, ten percent of final entries were cross-checked by a second researcher. The data sheet was checked for coding or data entry errors and cleaned. The data sheet was transferred to Statistical Package for the Social Sciences (SPSS version 26, SPSS Inc., Chicago, IL, USA) for analysis. Descriptive statistics are presented as means, frequencies, and proportions as appropriate.

## 3. Results

### 3.1. Demographic Characteristics of Participants

A total of 150 adult Samoans aged 20–77 years (M = 45.06 years) completed the questionnaire (Table 1).

The sample is somewhat representative of the Samoan population; however, we had a greater proportion of married participants. The latest census in Samoa (in 2016) reported that 51.5% of the population were male and 48.5% female [24]. Just over half (57%) were aged between 15 and 64 years of age, with 4.9% aged 64 years or above [24]. Most (59.3%) identified as being single, 34.4% married, 3.6% widowed, and 2.6% divorced [24].

### 3.2. Food Literacy Domains

#### 3.2.1. Plan and Manage

The majority of participants reported engaging in planning and managing behaviours most of the time or always, including: planning to include healthy food (87%, n = 126), budgeting money for food (87%, n = 125), planning meals ahead of time (75%, n = 109), and making a shopping list (69%, n = 99) (Table 2). A greater number of participants aged ≥35 years reported that they “always” plan to include healthy foods.

#### 3.2.2. Select

Within the “select” domain, one third of participants (32%, n = 47) did not know where to find the nutrition information on foods. The majority of participants (68%, n = 99) reported knowing where to find the nutrition information on foods, with most (43%, n = 41) reporting that they always used this to make purchase decisions (Table 2). Nine percent of participants (n = 14) reported that they knew where the nutrition information is but cannot use it due to it being in a different language. Sixty-four percent (n = 40) of females reported using the nutrition information always or most of the time, compared to 41% (n = 22) of males.

#### 3.2.3. Prepare

Over half of the participants reported always changing recipes to make them healthier (62%, n = 89) and feeling confident in cooking a variety of foods (67%, n = 96). Three quarters of participants (75.3%, n = 110; females: 79%, n= 61, males: 71%, n = 44) reported always or most of the time cooking meals at home (Table 2).

**Figure 2:**
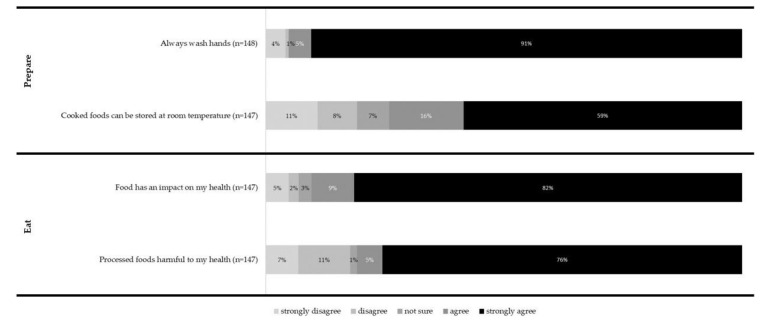
Frequency of activities related to prepare and eat domains.

Most participants (82%, n = 121; males: 86%, n = 55, females: 80%, n = 62) reported knowing how to store food to make it last longer. Almost three quarters (74%, n = 109) of participants either strongly agreed or agreed that cooked foods can be stored at room temperature overnight. Most participants (95%, n = 141) strongly agreed or agreed that they should wash their hands with soap and water before and after food preparation (Figure 2).

#### 3.2.4. Eat

**Figure 3:**
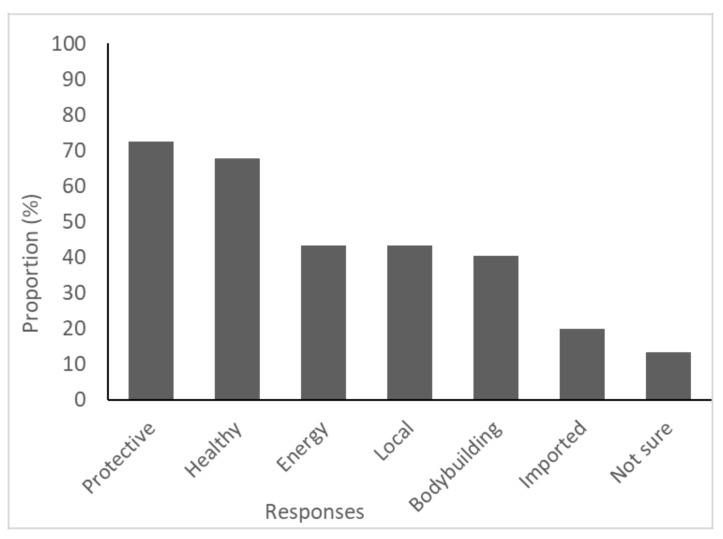
Frequency of responses to the question “can you identify the three food groups from the following?”.

Most participants (91%, n = 132) agreed, or strongly agreed, that food has an impact on their health. Slightly fewer participants (81%, n = 119) agreed or strongly agreed that eating processed foods high in salt and fat is harmful to their health (Figure 2). Ten percent (n = 15) of participants correctly identified all three food groups outlined in the Pacific Guidelines for Healthy Living [6], as protective foods, bodybuilding foods, and energy foods. Of the six provided options, protective (72%, n = 105; correct answer) and healthy food (68%, n = 99; incorrect answer) groups were the most frequently selected options (Figure 3).

**Figure 4:**
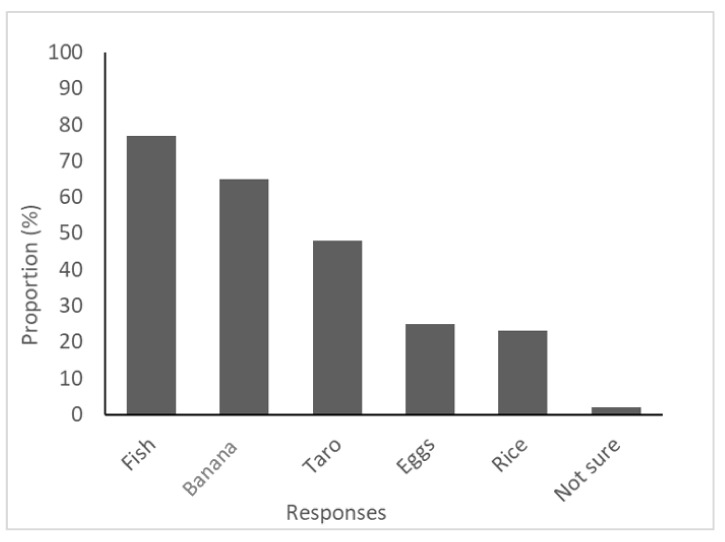
Frequency of responses to the question “can you identify the protective foods from the following?”.

When asked to identify the protective foods from the pictures, only two participants (1.4%) selected the two correct food items (banana and cabbage). Of the six options, cabbage was correctly identified by 83% (n = 121) of participants, whereas fish was incorrectly identified as a protective food by 77% (n = 112) of participants (Figure 4). Although most participants could not correctly identify all protective foods on the list, almost half (47%, n = 69) knew to include these foods at every meal.

## 4. Discussion

This descriptive, exploratory study aimed to describe food literacy in adult Samoans, in the context of the four domains of plan and manage, select, prepare, and eat. Nutrition transition has negatively impacted the health of Pacific Islanders [1]. Increased access to, and consumption of, highly processed foods has contributed to high rates of overweight, obesity, and DR-NCD’s in these populations, resulting in pressure on healthcare systems in this region. While we explored the domains of food literacy in a cohort of modest size, this work provides a productive starting point for further investigation of how this population makes food decisions (planning and managing, selecting, preparing, and eating foods) in a unique but dynamic food environment. We were able to identify some trends related to demographics, but further work is needed to confirm if these indeed exist in this population. However, this foundational work has the potential to assist with the design, implementation, and evaluation of public health interventions, and for policymakers in considering how the food environment can be designed to enable and support healthy, sustainable dietary behaviours in Samoa and the wider Pacific Islands region.

### 4.1. Plan and Manage

Planning and managing behaviours, including budgeting money for food and making feasible decisions to include healthy food, are essential in ensuring that individuals and households have access to enough, safe, nutritious, and culturally relevant food [25]. Results from our study show that the majority of participants reported engaging in planning and managing behaviours. There could be several reasons for this, including the need to budget for food, planning based on access to cooking amenities and facilities, opportunities for food procurement and transport of food home via public transport, and large household size. As almost three quarters of our sample indicated that they were married, these behaviours may reflect managing a household. It would be of interest to investigate if these behaviours are also seen at similar levels in other marital status groups.

The cost of food or perceptions of cost have been reported to influence food choice in Samoa [12,13], with one study reporting that perceptions of expensive foods were associated with decreased consumption [13]. The need to focus on cost may be associated with the proportion of income spent on food in Samoa. Results from the 2018 Household Income and Expenditure Survey show that Samoans spend approximately 45% of their total income on food [26]. It is also possible that planning to include healthy food is due to Samoans reporting a preference for local, traditional foods [12,14,15], which are often viewed as being healthier [12,15]. We did not explore the nature of these behaviours; however, this would be of interest in future work.

### 4.2. Select

In a food environment with increasing availability of processed foods, being able to identify what a food is, its nutrition composition, and to compare items to identify healthier options is important. Food labels are intended to provide consumers with access to reliable nutrition information to help them to make informed food choices [27]. Research has consistently showed a link between the use of nutrition labels and improved diet quality [28]. However, difficulties exist with the labelling of information and consumer interpretation [28]. We found that most participants reported being able to locate and use a nutrition information panel (NIP) when deciding what to eat. This is an interesting finding as previous research shows that Pacific Islanders (resident in New Zealand, Hawaii, and Utah) report difficulty understanding food labels [29,30]. Instead, Pacific Islanders showed a preference for simple nutrition messages, such as the New Zealand Heart Foundation tick and traffic light labels [30,31]. A potential reason that our results suggest that Samoans locate and use NIP’s may be the time that has elapsed between these studies and ours, that we did not ask for qualitative information on barriers/enablers to use, and/or the fact that what constitutes an NIP was not explicitly outlined in the questionnaire, resulting in responses that do not represent actual use.

Interestingly, almost 10% of participants reported that they could not read the nutrition information as it was in a different language. According to Samoa’s Food Safety and Quality Regulations (2017), a food label must, at least, be in the English or Samoan language. If the original label is not in either of these languages, the food product must be re-labelled or contain a supplementary label [32]. However, enforcement of these laws is unknown, so the prevalence of imprecise labels is not clear. Previous research examining food labels in five Pacific Island Countries (2013) found that whilst products may display a NIP, they are generally incomplete and omit key nutrients, including sodium and saturated and trans fats [33]. Given that only 3% of the processed products found in Samoa were produced in the country, it would be practical to investigate the quality and accuracy of food labels, to ensure trust and ease of use for consumers [33].

In the future, it would also be of interest to investigate the use of nutrition information between sexes. While we were not able to test for associations, we did find that a greater proportion of females reported using nutrition information to make a food choice. The literature suggests that females, in general, are more likely to prioritise health when choosing food and may therefore use the NIP to gauge the “healthiness” of foods [11]; however, there is no literature on this in Samoan populations. However, it is recognised that women tend to have greater involvement in food planning and meal preparation in Samoan culture and therefore have a greater responsibility for making appropriate food choices for the family [34], potentially reflecting their use of nutrition information. Future work should aim to investigate the use of nutrition information on foods, with a specific focus on associations with demographics.

### 4.3. Prepare

The third domain, “prepare”, describes the knowledge and skills required to construct a tasty, nutritious meal from available foods whilst following basic principles of food hygiene and handling [7]. Based on our findings, food safety practices in Samoa may be a public health concern. Although the majority of participants had knowledge of appropriate handwashing behaviours around meal preparation, knowledge of food storage was lacking. As stated in the World Health Organisation’s Five Keys to Safer Food Manual and further promoted in the Pacific Guidelines for Healthy Living, cooked food should not be stored at room temperature for over two hours [6,35]. Most participants felt that it was acceptable to store cooked foods at room temperature overnight. This could reflect a lack of knowledge and education on food safety practices, a lack of access to storage equipment, or a preference for traditional storage methods. Cultural norms in Samoa also suggest that some people believe food prepared to be eaten should be eaten at the time and not stored for later [6,12]. Overall, poor food storage practices increase the risk of foodborne illness [36] and highlight the importance of the Samoa National Codex Strategic Plan 2017-2021 in working towards strengthening national food safety systems and promoting compliance with food safety standards [37]. Future investigations should seek to further explore barriers and enablers of food safety and hygiene, including information sources, enforcement of regulation, and key behavioural motivators of consumers and vendors.

Our research into food preparation also shows that participants generally feel comfortable and confident cooking a variety of meals at home. This is likely because of the importance of food in Samoan culture, where food is believed to maintain connectedness and build harmonious relationships among family and community [38,39].

### 4.4. Eat

The final domain, “Eat”, describes the practicalities associated with eating for good health, including the ability to balance food intake, distinguish between healthy and less healthy foods, and knowledge of appropriate portion sizes and frequency of consumption [7]. Improving nutrition knowledge is a key element for increasing diet quality [40]. Our study investigated components of food knowledge. We found that the majority of participants agreed that food impacts health. Most participants could take this one step further, agreeing that processed foods high in salt and fat are harmful to their health. Previous research supports these findings, demonstrating that Samoan adults have a basic awareness of the association between diet and diet-related disease [30,41]. However, findings from previous studies highlight that this knowledge is limited, concluding a lack of personal ownership towards health and limited understanding of the nutrition composition of foods and how this affects the body [8,41].

Information on how to balance meals and include foods from all three food groups, energy foods, protective foods, and body building foods, can be found in the Pacific Guidelines for Healthy Living. We found that 10% of participants could correctly identify the three Pacific Island Food Groups (protective, bodybuilding, and energy) from a list and only two participants correctly identified the two protective foods from a six-item pictorial. Fish, however, was selected by 77% of participants as a protective food. Whilst this is incorrect, there may be a lack of understanding of food groups in this population. Within the Pacific Guidelines for Healthy Living, fish is categorised as a bodybuilding food. As fish, especially oily varieties, is well-known for its potential cardio-protective benefits [6,42], participants may see this as a “protective” food. Therefore, responses to this question may not be an accurate reflection of individuals’ knowledge of what constitutes a healthy diet. Despite the existence of this educational tool, the dissemination and understanding of the guidelines are unknown and may be a barrier to healthy dietary behaviours. Responses may instead reflect the challenges of dissemination of the Pacific Guidelines for Healthy Living [6], the naming of food groups, and that of making food choices in an environment in which highly processed foods are readily available.

An alternative explanation could be a lack of nutrition and health education in schools. Although there is a nutrition curriculum across both primary and secondary education in Samoa, compliance with the School Food Policy can be challenging [11,43]. Stakeholders involved in the development of Samoa’s dietary guidelines (2021) [44] could investigate current and future dissemination strategies to ensure that individuals and communities have access to reliable health information to equip them with the knowledge and understanding required to make appropriate nutrition decisions [45]. Future research should explore nutrition knowledge in more detail to understand key areas for improvement.

### 4.5. Limitations

There are several limitations to our work. Our questionnaire did not cover all components of food literacy due to a lack of validated tools for use in this population and because we were mindful of the potential burden on participants who consented to take part in this work. Our sample size was modest, potentially limiting the generalisability and transferability of our findings. Furthermore, our sample size and differences in response rate between questions and across domains limited our ability to undertake more analysis within the data set. We do not know why there were there slight variations in the number of participants who did not provide an answer for some questions, but this may be due in part to the length of the questionnaire. Participants were provided with directions at the start of the questionnaire, as per ethical approval, indicating that they could decline to provide an answer for any question, possibly indicating that some questions were viewed as being of sensitive content. As we used an intercept survey, in busy public spaces, we did not record the number of participants who declined to take part. While we attempted to recruit a range of participants, we had higher representation of participants 35–54 years of age; however, this is reflective of the Samoan population. Our sample had a larger proportion of married and fewer single (marital status) participants, which is not reflective of the Samoan population. As marital status may influence food behaviours, it would be of interest to investigate these components with a larger sample of participants identifying as single marital status. There is no validated tool to assess food literacy in this population; however, our data collection tool was developed to align with the components of the food literacy framework outlined by Vidgen and Gallegos (2014), which encompasses the domains of plan and manage, select, prepare, and eat. Our tool was developed with local input to ensure that it considered cultural values and language, and the Samoan environment. Our tool relied on self-report data, which incurs implications including self-reporting bias and social desirability bias [46].

## 5. Conclusions

Our study showcases a first glimpse into the food literacy behaviours of Samoan adults. This exploratory, descriptive study is among some of the first to explore food literacy with an interviewer-administered questionnaire and, to the authors’ knowledge, is the first to do so in a Pacific Island setting. We explored components of food literacy across the four domains of plan and manage, select, prepare, and eat. While exploratory in nature, it appears that there may be differences between sociodemographics (i.e., sex, age, marital status) and some behaviours in this population. Our findings highlight areas for further investigation, including investigating associations between demographic-related motivators (i.e., sex, age, marital status) for planning and managing behaviours, shop and market vendor compliance with legislative requirements of food labels, food safety and hygiene practices, and nutrition knowledge among population groups. Despite the novel nature of these findings, these are likely a useful contribution to the design of public health initiatives and, with further investigation, could be of use to policymakers.

## Figures and Tables

**Table 1:** Participant demographics.

Demographic Characteristic (Total Responses n)	Frequency	Total (%) *
Gender (n = 144)		
Male	65	45
Female	78	54
Fa’afafine ^	1	1
Age group (n = 150)		
20–34	23	15
35–44	58	39
45–54	38	25
55+	31	21
Marital status (n = 150)		
Single	18	12
Never married	7	5
Married	111	74
Divorced	7	5
Widowed	4	3
Prefer not to say	3	2
Employment status (n = 148)		
Work on plantation	12	8
Part-time employed	9	6
Full-time employed	68	46
Unemployed and looking for job	18	12
Unemployed and not looking for job	36	24
Prefer not to say	5	4
Highest level of completed education (n = 147)		
Did not attend school	0	0
Primary	28	19
Secondary/High school	88	60
Technical college/Vocational/Trade college	14	9
University	17	12

* Percentage totals may not total 100% due to rounding. ^ Fa’afafine (n = 1) (in traditional Samoan culture, Fa’afafine are biologically male at birth but encompass male and female gender traits [23]).

**Table 2:** Frequency of activities related to plan and manage, select, and prepare domains.

Food Literacy Domain	Food Literacy Behaviour (n = Number of Total Responses)	Never or Rarely n (%)	Sometimes n (%)	Most of the Time n (%)	Always n (%)
Plan and manage	Plan meals ahead of time (145)	8 (6)	28 (19)	35 (24)	74 (51)
	Make a list before you go shopping (144)	26 (18)	19 (13)	12 (8)	87 (61)
	Plan to include healthy food (143)	4 (3)	13 (9)	29 (20)	97 (68)
	Budget money for food (142)	13 (9.0)	4 (3)	12 (8)	113 (80)
Select	Use the nutrition information when deciding what to eat (121)	21 (17)	32 (26)	25 (21)	43 (36)
Prepare	Cook meals at home (146)	8 (5)	28 (19)	30 (21)	80 (55)
	Feel confident cooking a variety of foods (143)	11 (8)	36 (25)	21 (15)	75 (52)
	Change recipes to make them heathier (143)	12 (8)	42 (29)	25 (18)	64 (45)

## Data Availability

Data can be requested from the corresponding authors.
